# Structure of Apo- and Monometalated Forms of NDM-1—A Highly Potent Carbapenem-Hydrolyzing Metallo-β-Lactamase

**DOI:** 10.1371/journal.pone.0024621

**Published:** 2011-09-08

**Authors:** Youngchang Kim, Christine Tesar, Joseph Mire, Robert Jedrzejczak, Andrew Binkowski, Gyorgy Babnigg, James Sacchettini, Andrzej Joachimiak

**Affiliations:** 1 Midwest Center for Structural Genomics and Structural Biology Center, Biosciences, Argonne National Laboratory, Argonne, Illinois, United States of America; 2 Department of Biochemistry and Biophysics, Texas A&M University, College Station, Texas, United States of America; 3 The University of Chicago, Department of Molecular Genetics & Cell Biology, Chicago, Illinois, United States of America; 4 Argonne, Illinois, United States of America; Loyola University Medical Center, United States of America

## Abstract

The New Delhi Metallo-β-lactamase (NDM-1) gene makes multiple pathogenic microorganisms resistant to all known β-lactam antibiotics. The rapid emergence of NDM-1 has been linked to mobile plasmids that move between different strains resulting in world-wide dissemination. Biochemical studies revealed that NDM-1 is capable of efficiently hydrolyzing a wide range of β-lactams, including many carbapenems considered as “last resort” antibiotics. The crystal structures of metal-free apo- and monozinc forms of NDM-1 presented here revealed an enlarged and flexible active site of class B1 metallo-β-lactamase. This site is capable of accommodating many β-lactam substrates by having many of the catalytic residues on flexible loops, which explains the observed extended spectrum activity of this zinc dependent β-lactamase. Indeed, five loops contribute “keg” residues in the active site including side chains involved in metal binding. Loop 1 in particular, shows conformational flexibility, apparently related to the acceptance and positioning of substrates for cleavage by a zinc-activated water molecule.

## Introduction

The imminent threat posed by the recent discovery and dissemination of the plasmid encoded New Delhi Metallo-β-lactamase (NDM-1) gene (*bla*NDM-1) harbored by multiple pathogenic microorganisms has prompted the formation of a global scientific *corps d'armée* [1]. Biochemical and structural elucidation of NDM-1 facilitates the thorough mechanistic understanding required for a rational design of small molecule inhibitors specific to NDM-1 for co-administration with β-lactam antibiotics. The crystal structures of NDM-1 presented here reveal an open, enlarged and flexible active site that explains the observed extended spectrum activity of this zinc dependent β-lactamase.

One of the last lines of defense against multiple and extensively drug resistant infections is the carbapenem class of β-lactam antibiotics, which was developed to evade β-lactamase mediated resistance posed by aerobic as well as anaerobic pathogens. Unfortunately, the integrity of the “big guns” (meropenem, imipenem, doripenem, ertapenem) has become compromised by a number of β-lactamases with extended spectrum activity, that is, the ability to inactivate all classes of β-lactam antibiotics, including carbapenems [2].

β-Lactams are the most broadly used antibacterials world-wide due to their effectiveness at irreversibly inhibiting cell wall biosynthetic enzymes required for peptidoglycan recycling, and minimal toxicity in humans [3], [4]. The first β-lactam discovered—penicillin—inhibits the function of the D-Ala-D-Ala transpeptidase that links the peptidoglycan molecules in bacteria [4]. Simultaneously, cell wall hydrolases and autolysins continue to break down peptidoglycan crosslinks, resulting in cellular lysis and death. Since the discovery of penicillin, several classes of naturally occurring and semi-synthetic β-lactams have entered the clinic. Concomitantly, broad use of β-lactams as antibacterials applies a selective pressure that increases the reproductive success of pathogenic strains carrying evolved β-lactamase genes capable of combating our arsenal of β-lactam antibiotics.

The vast structural diversity designed into the semi-synthetic β-lactams evades β-lactamase mediated resistance by either preventing initial Michaelis complex formation, or by stabilizing transient intermediates that inhibit further β-lactam turnover. Carbapenems have proven to be the most effective broad-spectrum β-lactams, and their utility is generally reserved as a last line of defense against the toughest drug-resistant infections including MRSA [5], XDR-TB [6], and bacterial meningitis [7].

However, it appears that the “target” met the challenge. In the past several years, new pathogenic strains carrying carbapenemase genes have been documented in patients from India, Pakistan, Bangladesh and other countries [8], [9], [10], [11], [12], [13], [14]. Carbapenemases are members of class A (KPC, IMI/NMC, SME), class B (IMP, VIM, SPM) and D (OXA) β-lactamases, for review see references [15], [16], [17]. Class B β-lactamases depend on divalent cation metal cofactors for their activity, and are described as metallo-β-lactamases (MBLs) [15], [17], [18], [19]. Unlike serine β-lactamases, MBLs are not inhibited by the classic irreversible β-lactamase inhibitors such as clavulanic acid, sulbactam and tazobactam, but instead are inhibited by metal chelators such as EDTA and o-phenanthroline [15], [16], [19]. Thiol compounds such as 2-omega-phenylalkyl-3-mercaptopropionic acid [20] and N-(2-mercaptoethyl)-2-phenylacetamide [21] are also competitive inhibitors. However, thus far the therapeutic potential of these inhibitors has not been demonstrated.

MBLs have been found in widely distributed bacteria such as *Escherichia coli*, *Klebsiella pneumoniae* and *Acinetobacter baumannii* [14], [15], [22], [23]. VIM and IMP are the most frequently acquired subclasses of B enzymes [16]. MBLs show significant diversity of the active site, catalytic properties, and metal ion requirements and have been divided into three subclasses: B1, B2, and B3 [2], [15], [16]. Subclass B1 includes several chromosomally encoded enzymes BcII, *Bacillus cereus* [24], CcrA, *Bacteroides fragilis* [25], BlaB, *Chryseobacterium meningosepticum* [26], and transferable VIM, IMP, SPM, and GIM type enzymes [15], [27], [28]. Subclass B2 includes CphA [29] and ImiS [30] lactamases from the *Aeromonas* species and Sfh-I from *Serratia fonticola* [31]. Subclass B3 is represented by L1 from *Stenotrophomonas maltophilia* [32], [33], FEZ-1 from *Fluoribacter gormanii* [34], [35], GOB from *E. meningoseptica* [36], [37], CAU-1 from *Caulobacter crescentus* [38], [39], and THIN-B from *Janthinobacterium lividum* [40].

A new mobile subgroup B of MBLs was recently discovered and named New Delhi Metallo-β-lactamase (*bla*NDM-1). This multi-drug resistance gene was characterized initially in an isolate from Sweden that originated from New Delhi, India. Since then, *Enterobacteriaceae* isolates harboring the NDM-1 gene have been found in multiple areas of India, Pakistan, Bangladesh but also in the USA, Canada, China, Japan, and United Kingdom [8], [9], [10], [11], [12], [13], [14]. The emergence of *bla*NDM-1 in India and China has been linked to its spread on the IncL/M incompatibility plasmid group types [41]. A current survey of the New Delhi vicinity identified NDM-1 carrying isolates from drinking water and seepage [22]. NDM-1 has been reported to be a highly potent carbapenem-hydrolyzing, zinc-dependent MBL. When expressed in bacteria, it makes these bacteria resistant to a broad range of β-lactams. Currently, no inhibitor of NDM-1 approved for medical treatment is available. NDM-1 positive bacterial infections can only be treated with a few antibacterials, including colistin, tigecycline, fluoroquinolones, D-captopril, and polymyxin B [42]. In view of the emerging multidrug-resistant strains carrying NDM-1, the discovery of effective inhibitors is critical and urgent. Here we report hydrolytic activity of NDM-1 against selected β-lactams and carbapenems, the three high-resolution crystal structures of apo- and the first structure of monometalated form of NDM-1 derived from *K. pneumoniae*. Our results show that NDM-1 is unique among other MBLs due to its enlarged and flexible active site, and explain the observed extended spectrum β-lactamase activity.

## Results and Discussion

### Expression, purification and structure determination of NDM-1

The NCBI database lists a number of identical sequences to NDM-1 from *K. pneumoniae* [CAZ39946)] from a number of other organisms [BAJ76899 [*Stenotrophomonas maltophilia*], ADY00041 and ADP20459 [*E. coli*], AEA41876 [*Acinetobacter baumannii*], ADU02194 [*A. junii*], and ADP37377 [*Enterococcus faecium*]]. Identical sequence entries are found in other records where the N-terminal region is missing. The N-terminal region of the NDM-1 protein contains a signal peptide [1]–[28] according to the Phobius transmembrane topology and signal peptide prediction server [43] consistent with the possible cleavage site after residue 28. Several other MBLs (VIM-1-Ec, VIM-2-Pa, IMP-1-Ab and IMP-4-Kp) contain signal peptides. In contrast, the PSORTb subcellular localization tool predicts no signal peptide and no predictive localization of the enzyme [44]. The sequence alignment of several selected MBLs reinforces the presence of a signal peptide (Fig. S1)with possible localization to the periplasm.

We expressed in *E. coli* full-length recombinant *K. pneumoniae* NDM-1 β-lactamase, but because it expressed poorly and showed low solubility, we designed several constructs. These constructs were based on the NDM-1 homology models obtained using tools developed as part of the Protein Structure Initiative [45] and low sequence similarity MBLs with structures available in Protein Data Bank (PDB) IMP-1, PDB id 1DD6 [46], VIM-2, PDB id 2YZ3 [47] and VIM-4, PDB id 2WHG [48]. Three recombinant NDM-1 β-lactamases: full-length NDM-1, and two constructs NDM-1 Δ38 and NDM-1 Δ36NY, which showed improved solubility, were expressed and purified to homogeneity. Purified enzymes exhibited a single band on SDS-PAGE, indicating M_r_ = 28.5 and 24.5 and 24.8 kDa for NDM-1, NDM-1 Δ38, and NDM-1 Δ36NY, respectively.

### NDM-1 Structure

The three apoNDM-1 structures include two pairs of monomers A and B and one monomer A, and the structure of monometalated NDM-1 (mZnNDM-1 Δ36NY) includes four monomers (A, B, C, D), respectively in the asymmetric unit, providing nine crystallographically independent views of the molecule for NDM-1 Δ38, NDM-1 Δ36NY (monoclinic), NDM-1 Δ36NY (orthorhombic), mZn-NDM-1 Δ36NY (tetragonal) (Table 1). Full length NDM-1 and NDM-1 Δ36NY are monomers in solution as shown by size exclusion chromatography (Fig. S2). The model for NDM-1 Δ38 contains 226 residues (45–270) out of a possible 232 residues and 131 water molecules. The electron density maps obtained for the NDM-1 Δ38 structure allowed modeling of 226 residues of both A and B chains, except the 10 N-terminal residues including the three residues Ser-Asn-Ala left over from cloning, which are disordered and not included in the final model. The NDM-1 Δ38 models were refined against 2.0 Å data with a final R_work_ of 19.5% and R_free_ of 24.3% (Table 1). The structure of NDM-1 Δ36NY, which includes two chains each containing residues of 45–270 (A) and 44–270 (B), is very similar to that of NDM-1 Δ38 (rms difference between Cα atoms of comparing 452 residues of both chains is 0.21 Å), except for a few loop regions and an acetate molecule found in the active site in chain A. The two structures of NDM-1 Δ36NY obtained under different crystallization conditions and in different crystal packing are virtually identical (rmsd between Ca atoms of comparing 436 residues of both chains is 0.45 Å). Our NDM-1 Δ38 and two NDM-1 Δ36NY structures are also very similar to the recently published high-resolution structure of dizinc NDM-1 Δ29 [49], except for the N-terminal region (missing or disordered in our structures and some loop regions (see below)). The structure of mZnNDM-1 Δ36NY was obtained in the presence of 10 mM zinc chloride. The structure contains one zinc atom bound in the active site to the metal site 1 (Zn1) (see below). The metal bound structure is very similar to apo structures (for example, rmsd between Cα atoms of apoNDM-1 Δ38 comparing 218 residues of chain B of mZnNDM-1 Δ36NY is 0.46 Å).

**Table 1:** Summary of the NDM-1 crystallographic data.

Data collection statistics	NDM-1 Δ38 (39–270)	NDM-1 Δ36NY (37–270)	NDM-1 Δ36NY (37–270)	mZnNDM-1 Δ36NY(37–270)
Space group	P2_1_	P2_1_	I222	P4_3_2_1_2
Unit cell (Å)	*a* = 59.71*b* = 51.11*c* = 70.68*β* = 106.96	*a* = 59.76*b* = 50.86*c* = 70.72*β* = 106.98	*a* = 66.05*b* = 83.27*c* = 10.5.4	*a* = 97.94*b* = 97.94*c* = 187.55
Wavelength (Å)	0.9793	0.9793	0.9792	1.2825
Highest Resolution bin (Å)	2.03-2.00	2.39-2.35	2.34-2.30	2.31-2.27
Number of observed reflections	26835 (1377)^b^	16892 (709)^b^	12326(528)^b^	42642 (2066)^b^
*R*_merge_ (%)^a^	15.3 (55.7)^b^	14.4 (44.8)^b^	9.1(33.4)^b^	10.4 (57.0)^b^
Completeness (%)	95.0 (96.8)^b^	97.6 (80.9)^b^	95.5(84.3)^b^	99.1 (98.1)^b^
*I*/σ *I*	6.3 (2.5)^b^	5.9 (2.1)^b^	11.9(3.5)^b^	6.3 (2.0)^b^
**Phasing and Refinement**	**MR**	**MR**	**MR**	**MR**
Search model	Chain A of 2YZ3	Chain A of 3RKJ	Chain A of 3RKJ	Chain A of 3Q6X
Phasing resolution range (Å)	38.4-2.00	40.7-2.35	36.9-2.31	38.4-2.27
Refinement resolution range (Å)	38.4-2.00	40.7-2.35	36.9-2.31	38.4-2.27
*R*_cryst_ (%)	19.2	20.8	20.3	18.3
*R*_free_ (%)	24.3	28.3	26.0	23.6
Number of protein residues	464	464	237	928
Solvent molecules	262	170	61	324
Bond lengths (Å)	0.007	0.017	0.012	0.011
Bond angles (deg)	1.19	1.61	1.52	1.33
B-factors (Å^2^)	30.1	30.6	50.6	32.6
Protein main chain	25.83	28.62	46.60	30.08
Protein side chain	31.04	30.31	52.92	33.51
Solvent (Water)	38.49	34.58	47.28	37.46
Wilson B-factor (Å^2^)	25.89	24.97	36.45	30.32
Ramachandran Plot (%)^c^				
Preferred	97.4	98.0	97.8	99.5
Generously allowed	2.6	2.0	2.2	0.4
Disallowed	0	0	0	0.1
**PDB ID**	3RKJ	3RKK	3SBL	3SFP

a *R*_merge_ = 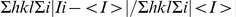, where *Ii* is the intensity for the *i*th measurement of an equivalent reflection with indices *h*, *k*, and *l*.

b Numbers in parentheses are values for the highest-resolution bin.

c Refined using PHENIX.

**Figure 1:**
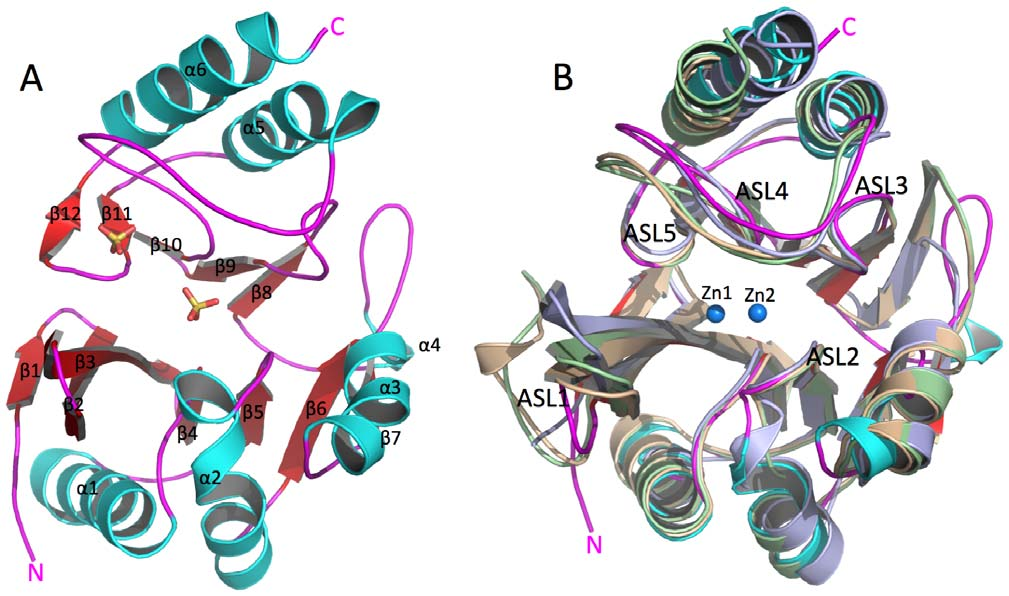
NDM-1 structure and comparisons with selected carbapenemases. **A.** Aerial view of active site (represented by two phosphate ions), the protein is made up of four layers, α/β/β/α, and forms a sandwich. Secondary structure elements and N- and C-termini are labeled. **B.** Comparison of *K. pneumoniae* NDM-1 (PDB id 3RKJ – magenta, N and C-termini are labeled) with three MBLs with carbapenemase activity *P. aeruginosa* VIM-2 (PDB id 2YZ3 - wheat), *P. aeruginosa* IMP-1 (PDB id 1DD6 - light blue) and *P. aeruginosa* VIM-4 (PDB id 2WHG - pale green). Loops contributing to the active site are labeled ASL1–5. Zn1 and Zn2 (blue spheres) are from the structure of VIM-2.

NDM-1 resembles the common β-lactamase fold, although it shows low sequence similarity to β-lactamases deposited in PDB (20–33% sequence identity). The protein is made up of four layers α/β/β/α and forms a sandwich. The core of NDM-1 consists of two β-sheets, one (N-terminal) is composed of seven antiparallel strands (β1–β7) and the other (C-terminal) is composed of five antiparallel strands (β8–β12). The N-terminal β-sheet is highly twisted (>100 degrees). The interaction between β-sheets is mainly hydrophobic. The seven connecting helices are located below (α1–α4 and 3_10_ helix 5) and above (α6–α7) the plane of the β-sandwich (Fig. 1A). The interactions between helices and β-sheets are hydrophobic but also involve several hydrogen bonds (Q96 with carbonyl of Y64, T98 with carbonyl of A92, Y229 with carbonyl of Leu209 and S232 with carbonyl of P187). Strands and helices are connected through flexible loops with the most prominent loop (residues 206–228) located above an ∼600 Å^3^ (see discussion) active site cavity containing a sulfate ion in monomer A and two sulfate ions in monomer B. The two β-sheets and four associated helices show a previously reported two-fold symmetry of the “ββββαβαβ” topological motif [24]. The symmetry is not ideal with one unit showing a “βββαβαβ” motif and the second unit “ββββαβαβαβ” is enlarged. The NDM-1 secondary structure is shown in Fig. 1A.

The search for structural homologs of NDM-1 using the DALI server [50] identified a number of closely related homologs. More than 400 entries were identified with a Z-score higher than 4. The closest structural homologs are MBLs (class B), followed by class A, C, and D β-lactamases. Over 1,200 β-lactamase superfamily members were also identified in 340 species showing highly diverse sequences. The top 250 structural homologs (Z-score>10) were clustered using CD-HIT [51] with a 90% sequence identity cut-off value resulting in 40 structure classes. Similar clustering was observed using 80% and 70% cut-off values. Representative structures from the 40 structure clusters were aligned based on the secondary structure (Fig. S3). The alignment reveals that the NDM-1 structure (and its MBL homologs) displays expansions in some loop regions, for example ASL1, ASL4 (Fig. S4, see below). The proteins belonging to the 40 structure clusters show several different catalytic activities (lactamase, oxidoreductase, hydroxyacylglutathione hydrolase, glyoxalase, nitric oxide reductase, parathion hydrolase, teichoic acid phosphorylcholine esterase). These enzymes utilize a broad range of substrates (β-lactams, hydroxyacylglutathione, nitric oxide, parathion, phosphorylcholine) suggesting that the lactamase fold seems highly adaptable and has evolved to support multiple functions and accept a wide range of substrates [15].

### Active site expansion in NDM-1 and implications for ligand binding

**Figure 2:**
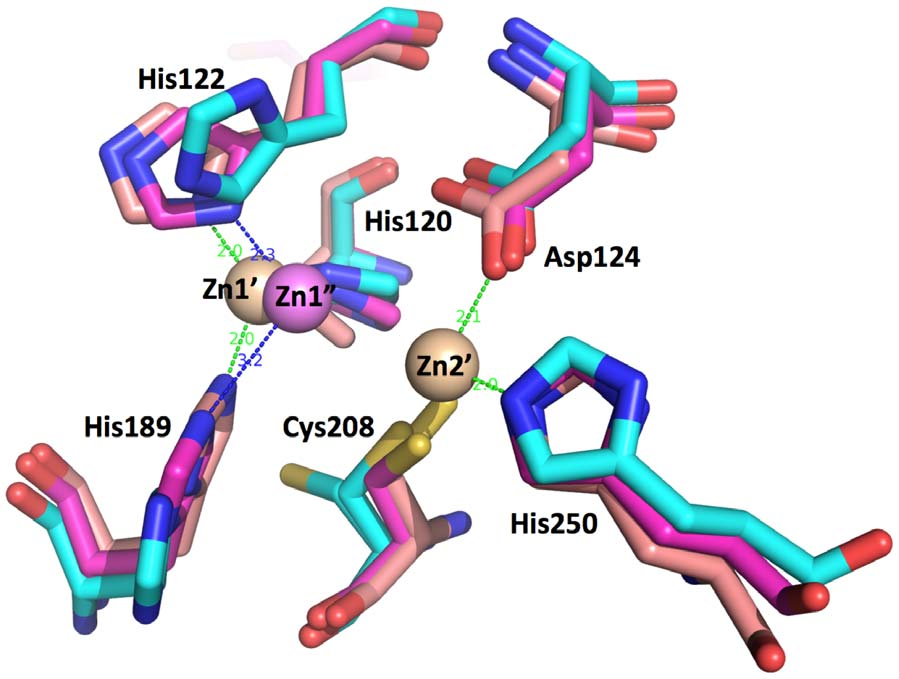
Active site comparisons of *K. pneumoniae* apoNDM-1 (PDB id 3RKJ) (aqua) vs monozinc NDM-1 (PDB id 3SFP) (violet) vs di-zinc NDM-1 PDB id 3Q6X [**49**] (wheat). Zn1′ and Zn2′ (wheat) are from the structure of di-zinc NDM-1 and Zn1″ is from the structure of monozinc NDM-1. Conformations of residues coordinating Zn1 (His120, His122 and His189) and Zn2 (Asp124, Cys208 and His250) are shown for all three structures.

**Figure 3:**
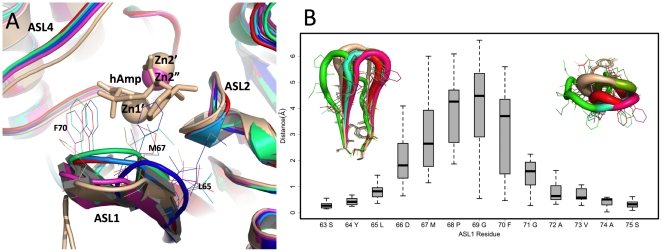
Flexibility of the active site loops. **A.** Structures of six NDM-1 molecules (identified using PDB ids) were aligned (PDB id 3RKJ (this work) molecule A - blue, PDB id 3RKJ (this work) molecule B - light blue, PDB id 3SBL (this work) molecule A - green, PDB id 3RKK (this work) molecule B – red, PDB id 3SFP (this work) molecule B – violet and PDB id 3Q6X [49] molecule B – wheat), hAmp is a hydrolyzed ampicillin and Zn1′ and Zn2′ are zinc atoms from the structure of NDM-1 (PDB id 3Q6X molecule B) and Zn1″ is from the structure of monozinc NDM-1. **B.** The structural variability at each residue position in the ASL1 loop is evaluated across six structurally unique loops shown in A. After a structural alignment of the entire molecules, the Euclidean distance between the residues' center-of-mass was measured between all pair combinations at each position. The results are summarized as a boxplot showing the median, quartiles, maximum and minimum distances for each residue. The aligned loops are shown in cartoon putty representation, with the loop radius proportional to residue B-factors (view facing active site, left; top-down view, right).

Active site template searches using ProFunc [52] identified the *B. fragilis* MBL (PDB id 1BMI) [53] as an active site match with a similarity score of 422.6 and E-value of 1.09×10^∧−10^. Seven active site residues in the *B. fragilis* MBL structure (His82, His84, Asp86, His145, Cys164, Asn176, His206) matched the corresponding residues in NDM-1 (His120, His122, Asp124, His189, Cys208, Asn220, His250) with an rmsd of 1.87 Å (over 13 atoms). Overall, the template identified 19 identical residues and 8 similar residues in the NDM-1/*B. fragilis* MBL active sites with local sequence identity of 52.8%. These residues form a solvent accessible surface that extends along the middle of the molecule between two β-sheets (Figs. 1, 2, S5). This well-defined hydrophobic and partly positively charged cavity is shaped by five active site loops (ASL1–5). From the bottom (as viewed in Figs. 1A, 2 and 3), two loops between strands β2 and β3 (residues 65–73, ASL1) and β5 and α2 (residues 118–124, ASL2) are contributing to form the base of the active site. At the top of the pocket, loops between β8 and β9 (residues 184–194, ASL3), β10 and α5 (residues 206–228, ASL4), and β11 and β12 (residues 248–255, ASL5) form the roof and walls of the active site (Fig. 1). In MBLs, these loops provide key conserved side chains for coordinating metal ions as well as proposed catalytic general acid/base. As evident from the NDM-1 structures, these residues appear to have well-defined conformations in the absence and presence of metal ions and this part of the active site is structurally well conserved (Fig. 2) (see below). Additional residues from these loops, in particular ASL1, seem to also participate in the positioning of ligands in the active site in an orientation suitable for hydrolysis of the β-lactam ring. This is evident from several crystal structures of MBLs obtained with ligands [26], [34], [46], [54], [55], [56]. MBLs, including NDM-1, show the longest ASL1 as compared with other β-lactamase superfamily members (Fig. S3). The alignment of the NDM-1 structure with the closest MBL structural homologs, di-zinc VIM-4 (PDB id 2WHG, Z-score 31.9, rmsd 1.9 Å), VIM-2 (PDB id 2YZ3, Z-score 11.2, rmsd 1.45 Å, between chain A of 3RKJ and chain A of 2YZ3), and IMP-1 (PDB id 1DD6, Z-score 11.7, rmsd 1.52 Å, between chain A of 3RKJ and chain A of 1DD6) reveals several unique features of the NDM-1 structure (Fig. 1). Region 162–176 shows a very different conformation with a short 3_10_ helix (α4) formed in the middle of the loop region (Fig. 1B). This region forms a β-strand in VIM-2, VIM-4 and IMP-1. NDM-1 and IMP-1 show the longest ASL1 loop (Figs. 1 and S1) but NDM-1 has a Phe insertion instead of Trp in this loop (Fig. S1). This smaller side chain may provide more flexibility to accommodate bulky substrates. In NDM-1, ASL4 shows a more open conformation and the C-terminal helix is quite shifted in comparison to VIM-2, VIN-4 and IMP-1. However, the most important difference between these MBLs structures is a significantly larger active site cavity. This region in NDM-1 provides the opportunity to accommodate large substrates. In VIM-2, VIM-4 and IMP-1, side chains from the ASL1 and ASL4 loops bridge over the active site, dividing it into sub-cavities [46], [47], [48], [53]. In contrast, the NDM-1 ASL1 and ASL4 loops are shifted outward considerably (Figs. 1B and 3) and do not close over the active site but instead leave the site much more open and accessible to potential ligands (Figs. 1B and 3).

**Figure 4:**
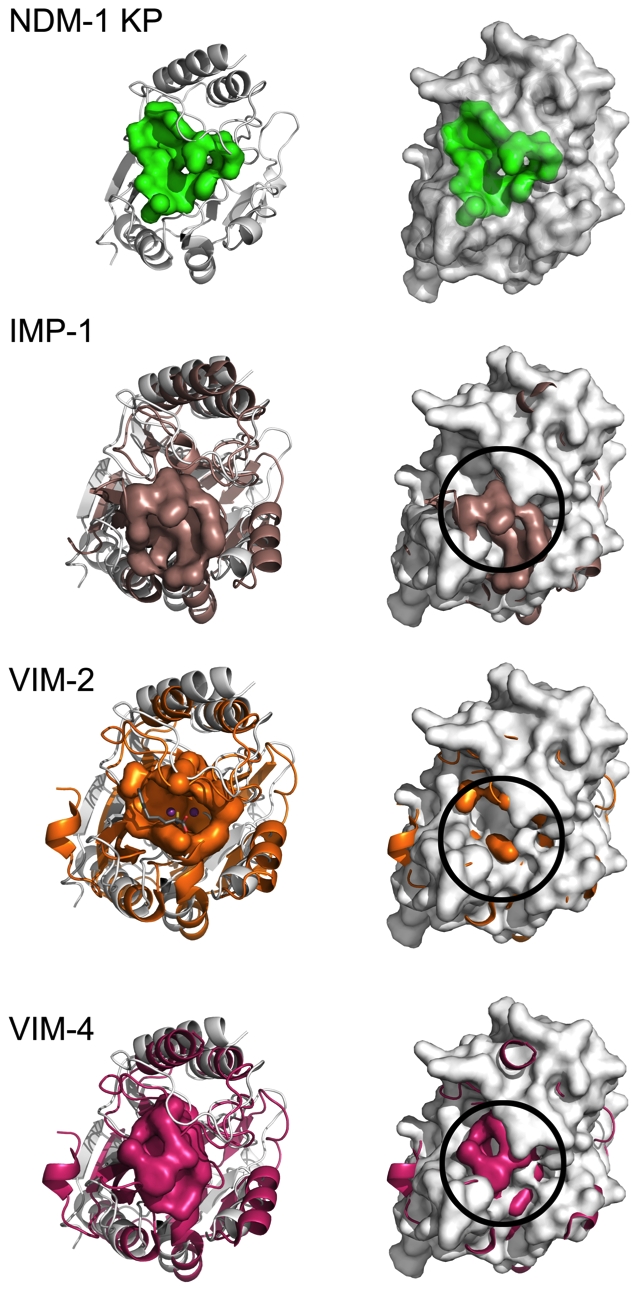
Active site expansion of NDM-1. The active site cavity comparisons of *K. pneumoniae* NDM-1 (gray) and three MBLs: IMP-1 (light brown), VIM-2 (orange) and VIM-4 (magenta). Superposition of the IMP-1, VIM-2 and VIM-4 molecules with NDM-1 reveal highly conserved structural arrangements. The most prominent variations occur between the catalytic sites (shown as surface representations), in which the side chains of IMP-1, VIM-2 and VIM-4 restrict the access to the active site by decreasing the volume of the cavity. The top row depicts NDM-1 and its active site surface (green) in secondary structure cartoon (left) and surface representation (right). The subsequent rows show NDM-1 structural alignments with IMP-1, VIM-2 and VIM-4. Each row highlights the IMP-1, VIM-2 and VIM-4 molecules' respective active sites, shown as colored surfaces. To highlight the greater accessibility to the NDM-1 active site, the aligned NDM-1 molecule is shown in surface representation (right). The obstructing interactions between ASLs restricting access to active site in IMP-1, VIM-2 and VIM-4 are observed as colored protrusions through the NDM-1 molecule and are highlighted for IMP-1, VIM-2 and VIM-4 with black circles.

There is a reduction in the volume of residues contributing to the active site (Ala121 in NDM-1, typically Phe or Trp in other MBLs; Tyr229 in NDM-1, typically Trp in other MBLs; sequence Ala72Val73 in NDM-1 in ASL1, which is typically Val or Phe in other MBLs; Phe70 in NDM-1 in ASL1 which is Trp in IMP-1 and related MBLs). There is also a reduction in the volume of residues contributing to the hydrophobic core. One indication of this volume reduction is an increase (15.2%) in alanine occurrence in NDM-1, which is nearly double the average alanine occurrence typically found in other proteins. These changes can contribute to the opening of the groove to solvent and can also increase the flexibility of structural elements. A larger active site would be more accessible to a broader range of antibiotics (or inhibitors) and thus provide an evolutionary advantage to bacteria. This can explain the observed enzyme promiscuity in accepting and hydrolyzing a broad range of β-lactams and carbapenems (Fig. S5). A more open active site would allow for many substrates to bind in an extended conformation along the elongated-shaped groove with the β-lactam ring positioned over the active site metal ions while the rest of the substrate could be further stabilized by the interaction with side chains projecting from flexible loops ASL3, ASL4, and ASL5 (Figs. 3, 4 and S5).

To investigate the structural implications of residue substitutions near the active site cavity, we performed a surface analysis on IMP-1 (PDB id 1DD6), VIM-2 (PDB id 2YZ3), VIM-4 (PDB id 2WHG), and NDM-1. For each protein, solvent accessible cavities were identified [57]. NDM-1 has the largest cavity (surface area = 412.4 Å^2^; volume = 591.3 Å^3^) followed by IMP-1 (surface area = 339.5 Å^2^; volume = 303.1 Å^3^), VIM-2 (surface area = 223.9 Å^2^; volume = 140.1 Å^3^), and VIM-4 (surface area = 112.4 Å^2^; volume = 45.3 Å^3^). The active site cavity for each protein is shown in Fig. 4 (green). It should be noted that the VIM-2 structure has a bound ligand in the cavity, which may affect the apo cavity structure and volume. However, even in a bound state the volume is considerably less than the NDM-1 cavity. To highlight the differences between the cavities, the molecules were superimposed onto the NDM-1 molecule. It is evident that the expanded cavity volume in NDM-1 is due to residue substitutions occurring in the surrounding loops. Fig. 4 shows the protruding residues in both stick and surface rendered forms.

In our three apo and one monozinc structures, there are nine independent representations of NDM-1 molecules with different crystallization conditions and packing environments. In these structures, most active site loops show very small conformational changes with the exception of ASL1 (Figs. 1B and 3) and to some extent ASL4. These are key loops contributing to the expansion of active site accessibility. Zhang and Hao, based on the comparison of their NDM-1 structure with other MBLs, hypothesized that the ASL1 loop may be flexible [49]. Our data clearly demonstrate that this is indeed true. As shown in Fig. 3B, ASL1 shows the ability of assuming several alternative conformations that may be important for binding ligands.

### Metal binding

NDM-1 has both β-lactamase and carbapenemase activities that are zinc-dependent [22], [49], [58], [59], this work. Three of our apoNDM-1 structures are zinc-free and are the first structures of a wild-type apo-enzyme in subclass B1. Interestingly, the second crystal form (PDB id 3RKK) was obtained in the presence of 5 mM zinc acetate. No zinc is found in the structure and only an acetate ion can be modeled in the active site interacting with His250. Because of the location, the architecture and sequence conservation of the active site in NDM-1 are very similar to the metal binding sites of other β-lactamases. The active site similarity (presence of principal zinc-binding motif (HaHqD)) allows us to unambiguously identify all residues involved in zinc coordination. The active site template search identified all residues that are involved in metal binding in other MBLs of B1 subclass. It is predicted that NDM-1 would bind two zinc ions. Zinc 1 would be coordinated by His120, His122 and His189 and zinc 2 by Asp124, Cys208 and His250. Indeed, zinc was found bound to the Zn1 site in the crystal structure of NDM-1 obtained in the presence of zinc chloride. All four monomers in the asymmetric unit contain zinc atoms bound to the Zn1 site (His120, His122 and His189), therefore confirming NDM-1 as a member of subclass B1 of MBLs [49], [59] and showing that Zn1 is a higher affinity site, as predicted for other MBLs [60], [61]. This zinc atom is shifted approximately 0.8 Å as compared with the di-zinc NDM-1 structure and shows somewhat longer Zn-His distances (Fig. 2) [49]. The zinc coordinating residues are provided by ASL2, ASL3, ASL4, and ASL5. ASL2 contributes residues coordinating both zinc ions. In apoNDM-1 structure conformations of some of these residues are very similar to zinc-bound states (His120, Asp124, and His250), while others show somewhat different side chain orientations (His122, His189, and Cys208) (Fig. 2). Cys208 exists in two orientations in apo-state, one is similar to the zinc bound state and the other is rotated about 45 and 90 degrees in chain A and B, respectively. It has been noticed that MBLs are sensitive to metal chelators and it was suggested that they could bind zinc with lower affinity [15], [16], [29], [60], [62]. The range of affinities for zinc reported for MBLs vary from micromolar to millimolar. All these residues are part of the “keg” structure of the active site.

The NDM-1 active site is located on its surface and is fully accessible to solvent. In the apoNDM-1 structure, two water molecules are coordinated by zinc-binding side chains, including Asp124, His122, and His250. In MBLs, the metal ions may have a dual role in catalysis. One zinc ion activates a metal-bound water molecule to perform a nucleophilic attack on the β-lactam carbonyl. The second zinc ion binds and polarizes this carbonyl group. A negatively charged carboxyl group of a zinc-bound Asp residue is involved in the activation of the water molecule. During the reaction, a tetrahedral intermediate is formed, followed by the delivery of a proton from a general acid/base residue (possibly Asp or another water molecule) to the lactam nitrogen and the cleavage of the lactam bond. All required residues are present in NDM-1 and therefore this enzyme should follow the general MBL catalytic mechanism involving a hydrolytic water molecule activated by a metal ion. In the mZnNDM-1 Δ36NY structures, there are several water molecules near the zinc atom. Monomer C contains tightly coordinated water (H-bond distance 2.8 Å) that may correspond to the position of the catalytic water molecule involved in β-lactam hydrolysis. Conserved Asp223 was proposed to function as the general acid/base during catalysis suggesting that conserved Thr190 (or Ser), which is centrally positioned on the loop overhanging the active site, may play an important role in substrate recognition and transition-state stabilization [61], [63]. It is easy to envision this loop undergoing movements upon substrate binding. The structure of zinc-free NDM-1, along with the previously reported structures, provides important insight into the conformational changes associated with the metal binding properties of NDM-1.

### Steady State Kinetics

NDM-1 kinetic parameters obtained using NDM-1 Δ38 are summarized in Table 2. NDM-1 is an extended spectrum zinc dependent β-lactamase capable of hydrolyzing nearly all classes of β-lactams, impairing the ability to treat life-threatening infections with intravenously and orally available carbapenems.. Carbapenem substrates appear to be the most specific class of β-lactams for NDM-1. However, the specificity constants for cephamycin and penem substrates deviate by less than an order of magnitude, indicating that the evolved promiscuity is related to the relaxed the substrate specificity of NDM-1. The observed lack of well-defined substrate specificity has previously been observed for the MBL NDM-1 homolog GIM-1 [28], [64].

**Table 2:** Steady state kinetic parameters of NDM-1 Δ38. with a selected set of β-lactam antibiotics (see Materials and Methods for details and Figure S6).

β-lactam antibiotic	β-lactam Class	K_M_ (µM)	Error	% Error	Vmax (µM s^−1^)	Error	% Error	*kcat* (s^−1^)	kcat/K_M_ (s^−1^/µM)
Biapenem	carbapenem	130	12	9	2.6E-03	5.8E-05	3	49.8	0.38
Imipenem	carbapenem	134	12	9	2.5E-03	7.8E-05	3	64.9	0.48
Tebipenem	carbapenem	69	7	10	2.8E-03	4.3E-05	3	39.0	0.57
Nitrocefin	cephalosporin	3	0.6	20	5.6E-03	2.2E-05	5	12.5	4.18
Cefoxitin	cephamycin	95	20	21	5.8E-03	3.4E-05	7	13.2	0.14
Faropenem	penem	99	16	16	4.5E-03	4.2E-05	8	14.7	0.15

The carbapenems; imipenem, biapenem and tebipenem have very similar kinetic constants likely due to their structural similarity. Tebipenem, although turned over less quickly (*kcat* = 0.39 s^−1^), has relatively high specificity, with a nearly two fold tighter Michaelis complex (K_M_ = 69 µM) relative to other carbapenems. The majority of the Michaelis constants falls between 50 µM and 150 µM, and includes penem, carbapenem and cephamycin classes of β-lactams (Fig. S6A–F).

Substrates that are most efficiently (*kcat*/K_M_) turned over in general have extended hydrophobic characteristics that complement the linear and hydrophobic nature of the NDM-1 active site cavity (Fig. S5). The primary hydrophobic arrangement is contributed by residues Leu65, Met67, Pro68, Val73, Gly69, Phe70, and Val73 of ASL1, which contour the flexible roof of the active site (Figs. 3, 4 and S5). In addition, alkyl moieties of Leu209, Ile210, Lys211, Asp212, Lys214, Ala215, Lys216, and Asn220 side chains of ASL4 give the distal region and base of the active site a partially hydrophobic surface area, while simultaneously maintaining hydrogen bond capability. These residues contribute to the “keg” set of side chains that constitute the active site. The degrees of freedom of the aforementioned residues serve to increase the flexibility of the active site, thereby increasing promiscuity, but may do so at the expense of Michaelis complex formation for most substrates.

In accordance with previous kinetic characterizations of metallo-β-lactamases GIM-1 [28], [64], IMP-1 [65], VIM-2 [66], and NDM-1 [42], kinetic constants could not be calculated for the suicide inhibitor clavulanic acid or the monobactam aztreonam. Although not yet implemented, this observation signifies the potential of treating NDM-1 harboring pathogenic bacteria with aztreonam or other structurally similar monobactams in combination with a new NDM-1 inhibitor.

### Mechanistic and drug discovery implications

It is likely that NDM-1 has evolved to have a more open and flexible active site, to be able to inactivate the highly decorated β-lactam antibiotics that have progressed over the years from relatively simple (penicillin) to larger and more complex compounds with new added substituents. It has been suggested that evolution of MBLs has enabled these enzymes to hydrolyze many compounds that interfere with key bacterial pathways, and is linked to increased flexibility and improved catalysis [61], [63], [67].

The X-ray structures of NDM-1 presented herein provide a structural foundation for the corroboration of the proposed reaction mechanism of NDM-1 and drug design. It seems that there are three distinct states of the NDM-1: metal free, singly metalated and doubly metalated. All these states could be targeted for structure-based inhibitor design. Analysis of structures and previously reported kinetic and spectroscopic studies on MBL enzymes allows us to propose a refined mechanism of catalysis for this antibiotic resistant lactamase. NDM-1 would bind a zinc atom to the Zn1 site and a second zinc to the Zn2 site. The binding of the second zinc could be cooperative as proposed for some MBLs. This would organize the residue in the active site for productive catalysis. The key step in catalysis for NDM-1 is likely recognition of the ligand by side chains in the cavity adjacent to the Zn1 site. Only ligands conforming to the NDM-1 site would bind. Next, the β-lactam ring of the ligand coordinates to Zn1, expanding its coordination number from four to five and activating it for a nucleophilic attack. It is clear that the ASL1 plays a key role in positioning the β-lactam ring over the dimetalated site. Deprotonation of the metal-bound water molecule by Asp124 to form a nucleophilic hydroxide moiety is consistent with the postulated p*K*_a_ of the zinc-bound water molecule. Once the zinc-bound hydroxide is formed, it can attack the activated carbonyl carbon of the substrate, forming a transition-state complex [61], [63], [68], [69]. In the dimetalated site, the second metal ion likely coordinates the β-lactam oxygen in a bridging fashion of the substrate. Asp124 may provide a proton to the penultimate amino nitrogen, similar to that observed for DapE [70], returning it to its ionized state thus facilitating product release. Here again the flexibility of ASL1 is important to release the product. Once the products are released, a water molecule bridging the two metal ions is replaced. Recent mechanistic studies of MBL showed that Zn2 is the only metal ion capable of stabilizing an anionic intermediate that accumulates during β-lactam hydrolysis, in which the C–N bond has already been cleaved. Conserved Asp124 would provide a proton to complete product release.

Kinetic studies with NDM-1 show that the K_M_ for biapenem and imipenem were 130 µM and 134 µM, respectively (Table 2, Fig. S6C,D), which are significantly above the minimum inhibitory concentrations (MICs) against most pathogenic microorganisms (<2 µg/mL or 6 µM) including *Klebsiella* spp. and *E. coli*, and *Pseudomonas aeruginosa* [71], [72], each of which has been found to harbor the NDM-1 gene [73]. Pharmacokinetic analysis demonstrates that human serum levels of biapenem were maintained at above the aforementioned MICs in young and elderly adults in a dose dependent manner [74]. Unlike other carbapenems, biapenem is stable to human renal dihydropeptidase-1 (DHP-1), eliminating the need to co-administer the DHP-1 inhibitor cilistatin [75]. These studies suggest that the orally available carbapenem, biapenem, could be of potential use to treat patients with NDM-11 linked bacterial infections because theMICs and human serum concentrations are far below the KM of biapenem for NDM-1. Therefore, next generation β-lactams used to fight NDM-1 linked bacterial infection may be derivatives of carbapenems like biapenem. However, given the broad specificity and likelihood of compensatory mutations, novel metallo-β-lactamase inhibitors will be required.

## Materials and Methods

### Protein cloning, expression, and purification

The ORF NDM-1 gene from *Klebsiella pneumoniae subsp. pneumoniae* MGH 78578 was synthesized chemically and initially cloned into vector pUC57. The full length NDM-1 and several N-terminal deletion constructs were subsequently amplified with KOD DNA polymerase using conditions and reagents provided by Novagen, Madison, WI and cloned into the pMCSG7 according to the ligation-independent procedure [76], [77] and transformed into the *E. coli* BL21(DE3)-Gold strain (Stratagene), which harbors an extra plasmid (pMgk) encoding one rare tRNA (corresponding to rare Arg codons, AGG and AGA). These constructs provided a system to produce a fusion protein containing an N-terminal His_6_-tag followed by a TEV protease cleavage site and a target protein (pMCSG7). To produce the protein, the bacterial culture was grown at 37°C, 200 rpm in enriched M9 medium [78] until it reached OD_600_ = 1.0. After air-cooling it down at 4°C for 60 min, NDM-1 expression was induced by 0.5 mM isopropyl-β-D-thiogalactoside (IPTG). The cells were incubated overnight at 18°C, harvested and resuspended in lysis buffer (500 mM NaCl, 5% (v/v) glycerol, 50 mM HEPES pH 8.0, 20 mM imidazole, and 10 mM β-mercaptoethanol). Cells were disrupted by lysozyme treatment (1 mg/ml) and sonication, and the insoluble cellular material was removed by centrifugation. The native NDM-1 protein was purified from other contaminating proteins using Ni-NTA affinity chromatography and the AKTAxpress system (GE Health Systems) with the addition of 10 mM β-mercaptoethanol in all buffers as described previously. This was followed by the cleavage of the His_6_-tag using recombinant His_6_-tagged TEV protease and an additional step of Ni-NTA affinity chromatography was performed to remove the protease, uncut protein, and affinity tag. The pure protein was concentrated using Centricon (Millipore, Bedford, MA, USA) in 20 mM HEPES pH 8.0 buffer, 250 mM NaCl, and 2 mM dithiothreitol (DTT). Protein concentrations were determined from the absorbance at 280 nm using a molar absorption coefficient (e_280_ = 28,500 M^−1^ cm^−1^) calculated by using the method developed by Gill and Hippel [79]. The concentration of NDM-1 Δ38 and NDM-1 Δ36NY samples used for crystallization was ∼40 mg/mL. Individual aliquots of purified NDM-1 Δ38 and NDM-1 Δ36NY were stored in −80°C until needed. The full-length protein expressed rather poorly at ∼2 mg/L of culture and it could only be concentrated to ∼8 mg/mL. Truncation of the first 38 residues of NDM-1 yielded a much more stable protein construct (NDM-1 Δ38) with the expression yield of soluble protein, ∼100 mg/L culture that can be concentrated up to 150 mg/mL.

### Protein crystallization

Native NDM-1 as well as several mutants, including NDM-1 Δ38 (the first 38 N-terminal residues deleted) and NDM-1 Δ36NY (the first 36 N-terminal residues deleted followed by Q36N and Q37Y mutations), were screened for crystallization conditions with the help of the Mosquito liquid dispenser (TTP Labtech, Cambridge, MA, USA) using the sitting-drop vapor-diffusion technique in 96-well CrystalQuick plates (Greiner Bio-one, Monroe, NC, USA). For each condition, 0.4 µL of protein (40 mg/mL) and 0.4 µL of crystallization formulation were mixed; the mixture was equilibrated against 135 µL of the reservoir in the well. Some protein was prepared in the presence of 5 mM zinc acetate or 5 mM zinc acetate and 20 mM citrate. Several commercially available crystallization screens were used including: MCSG-1–4 (Microlytic Inc. MA, USA) and Index (Hampton Research, Aliso Viejo, CA, USA) at 16°C and 4°C. Microcrystals were obtained under several conditions. Crystals of the zinc-free form of NDM-1 Δ38 and NDM-1 Δ36NY in the presence of 5 mM zinc acetate were grown at 16°C by vapor diffusion in sitting drops containing 0.4 µL of precipitant solution and 0.4 µL of 40 mg/mL of NDM-1 Δ38 or NDM-1 Δ36NY with 5 mM zinc acetate. The crystals grew within five days and reached sizes of approximately 0.100 mm×0.020 mm×0.010 mm^3^. The best crystals appeared at 16°C under the condition of 0.17 M ammonium sulfate, 25.5% (w/v) PEG4000 and 15% glycerol, which corresponds to condition G6 from the MCSG-3 screen. These crystals had dimensions of 100×25×10 microns^3^ and diffracted to 1.9 Å using the 19-ID minibeam [80]. The crystals of NDM-1 Δ38 belonged to the primitive space group P2_1_ with unit cell parameters *a* = 59.71 Å, *b* = 51.11 Å, *c* = 70.6 Å *β* = 106.96°. The NDM-1 Δ36NY crystals were also P2_1_ with a similar unit cell dimension of *a* = 59.76 Å, *b* = 50.86 Å, *c* = 70.72 Å, *β* = 106.98°. The asymmetric unit contains two molecules with a V_m_ value of 2.1 Å^3^/dalton (solvent content 41.5%). Data collection was carried out on the 19-ID beamline of the Structural Biology Center at the Advanced Photon Source according to the procedure described previously [81]. Data for the NDM-1 Δ38 and the NDM-1 Δ36NY crystals were collected to 2.0 Å and 2.35 Å at a wavelength of 0.9792 Å from the single crystals using a ∼20 micron mini-beam and were processed using HKL3000 [82] (Table 1). The third crystal was obtained from NDM-1 Δ36NY co-crystallizing with 10 mM aztreonam in the condition containing 1.8 M ammonium citrate dibasic, and 0.1 M sodium acetate trihydrate pH 4.6. The I-centered orthorhombic crystal (I222) of approximately 0.2×0.1×0.07 mm, a broken piece from a big multiple congregate, diffracted beyond 2.0 Å, though they are multiple with cell dimensions of *a* = 66.05 Å, *b* = 83.27 Å, *c* = 105.4 Å. The data were collected using ∼75 micron beam. Crystals of mZnNDM-1 Δ36NY were obtained in the presence of 10 mM ZnCl_2_ in the presence of 0.17 M ammonium sulfate, 25.5% (w/v) PEG 4000, 15% (v/v) glycerol and 20 mM citrate and belong to tetragonal space group P4_3_2_1_2, with unit cell dimensions of *a* = 97.94 Å, *b* = 97.94 Å, *c* = 187.55 Å. These crystals diffracted X-rays to 2.2 Å (Table 1).

### Size exclusion chromatography

The molecular weight of native NDM-1 protein in solution was determined by size-exclusion chromatography using a Superdex 200 GE Healthcare 16/60 column. The column was calibrated with aprotinin (6.5 kDa), carbonic anhydrase (29 kDa), conalbumin (75 kDa), catalase (232 kDa), and thyroglobulin (669 kDa). The separation was carried out at 22°C at a flow rate of 2.0 mL/min. The calibration curve of Kav versus log molecular weight was prepared using the equation Kav = (Ve−Vo/)/(Vt−Vo,), where Ve = elution volume for the protein, Vo = column void volume, and Vt = total bed volume. Size exclusion chromatography indicates a protein monomer (Fig. S2).

### Data collection

An X-ray diffraction data set extending to 2.0 Å resolution was collected at the Structural Biology Center 19-ID beamline with the 20×20 µm mini-beam at the Advanced Photon Source, Argonne National Laboratory using the program SBCcollect. The crystal was pre-cooled in liquid nitrogen and exposed for 3 sec. per 1.0° rotation of omega with the crystal to a detector distance of 280 mm at 100 K using 0.9792 Å X-rays. The complete data were recorded on an ADSC 315r CCD detector scanning 185° on omega until the crystal was severely decayed. The second crystal form of NDM-1 Δ36NY was prepared in the presence of 5 mM zinc acetate and diffracted beyond 2.35 Å and a data set was collected similarly at the same beamline. The third crystal form produced by co-crystallization with aztreonam diffracted beyond 2.0 Å, however, the data were good only to 2.30 Å, because the crystal was multiple and decaying during the data collection. The data were collected by the similar procedures. For the mZnNDM-1 Δ36NY crystal, the energy was set to 1.2825 Å to exploit anomalous signal from zinc atoms. All of the diffraction data were integrated and scaled with the HKL3000 suite [82]. The processing statistics are given in Table 1.

### Structure solution and refinement

The structure of NDM-1 Δ38 was determined by molecular replacement with the native data using MOLREP [83] within the HKL3000 software suite and BALBES [84] (R-factor of 42.2%, correlation coefficient score of 0.634), as described previously. Coordinates of MBL (PDB id 2YZ3) from *P. aeruginosa*, which exhibits ∼33% identity with NDM-1 from *K. pneumoniae*, were used as a search model. Further extensive model building was performed manually in COOT [85], while crystallographic maximum likelihood refinement with TLS groups [86] for two protein chains were refined by PHENIX.refine [87]. The structure of the two protein chains each containing residues 45–270 was refined to final R and R_free_ factors of 0.19 and 0.24, respectively. The final model is characterized by a 0.007 Å rmsd from ideal bond lengths. 97.8% of the residues occupy the most favored areas of the Ramachandran plot according to the MOLPROBITY validation results [88]. The 2.35 Å structure from the second crystal with zinc acetate was determined by molecular replacement using the first structure as a search model on HKL3000 and refined using COOT and PHENIX.refine. The final refined model of the two protein chains (residues of 44–270 and 45–270) converged to the R and R_free_ values of 0.21 and 0.28, respectively. The two protein structures are very close in details (rmsd of 0.21 Å with 452 Ca atoms) except that the second structure contains an acetate molecule in the active site. The structure was refined to a final model after several rounds of COOT and PHENIX.refine steps. Similarly, the third structure obtained from the co-crystallization with aztreonam was also determined to 2.31 Å by molecular replacement using the same search model (3RKJ). The final refined structure includes one chain with residues 41–269 and has an R_work_ of 20.6% and R_free_ of 27.0% with a good stereochemistry. Although the antibiotic molecule was not present in the structure, there were subtle changes found in the structure. For the NDM-1 Δ36NY crystal obtained in the presence of zinc chloride (mZnNDM-1 Δ36NY), the data were collected near the zinc absorption peak energy (1.2825 Å). However, zinc anomalous diffraction signal was weak and structure could not be solved. Instead, molecular replacement with the structure of chain A of 3Q6X as a search model yielded the structure using HKL3000. The structure contained a single zinc bound NDM-1 Δ36NY in all four monomers in the asymmetric unit. The subsequent number of cycles of refinement by PHENIX.refine and COOT finalized the structure with R_work_ of 18.1% and R_free_ of 23.6% with a good stereochemistry. The final refinement statistics for all three structures are presented in Table 1.

### Steady State Kinetics

Steady state kinetic parameters were calculated for NDM-1 Δ38 β-lactamase substrates by directly monitoring initial velocities as the appearance or disappearance of the respective β-lactam antibiotic chromophore over time; Nitrocefin (λ = 486 nm, ε = 20,500 M−1 cm−1), Imipenem (λ = 298 nm, Δε = 9,035 M^−1^ cm^−1^), Tebipenem (λ = 300 nm, Δε = 6,850 M^−1^ cm^−1^), Biapenem (λ = 295 nm, Δε = 7,020 M^−1^ cm^−1^), Cefoxitin (λ = 262 nm, Δε = 5,382 M^−1^ cm^−1^), Faropenem (λ = 306 nm, Δε = 2,662 M^−1^ cm^−1^) (Fig. S6). All experiments were performed in 10 mM HEPES pH = 6.75, 250 mM NaCl, 100 µM ZnCl_2_. The final concentration of NDM-1 was 10 nM. Reactions were performed in UV transparent Costar 96 well plates and monitored with the Thermo Scientific Multiskan Go plate reader. Initial velocities were fit to the Michaelis-Menten Equation using KaleidaGraph 4.0.

### PDB accession code

The atomic coordinates and structure factors have been deposited in the PDB with accession code 3RKJ, 3RKK, 3SBL and 3SFP.

## Supporting Information

Figure S1 **Sequence alignment of selected MBLs.** Amino acid sequence of *K. pneumoniae* NDM-1 was aligned with several MBLs (IMP-1 from *Acinetobacter baumannii* (gi110350569), IMP-4 from *K. pneumoniae* (gi|110350569), MBL from *B. fragilis* (gi|22091056), MBL-2 from *B. cereus* ATCC 10876 (gi|229191531), VIM-1 protein from *Enterobacter cloacae* (gi87158436), MBL VIM-11 from *P. aeruginosa* (gi|49035769), VIM-2 from *P. aeruginosa* (gi|126571829)) using ClustalX. The ‘T’ denotes turns. The blue boxes denote residues with >0.7 similarity. The Figure was prepared using ESPript 2.2 (http://espript.ibcp.fr/ESPript/ESPript).
(TIF)

Figure S2 **Size exclusion chromatography of native NDM-1 protein using a Superdex 200 GE Healthcare 16/60 column (blue circle).** The separation was carried out at 22°C at a flow rate of 2.0 mL/min. The column was calibrated with molecular weight standards (red circles) aprotinin (6.5 kDa), ribonuclease (13.5 kDa), carbonic anhydrase (29 kDa), ovalbumin (43 kDa), conalbumin (75 kDa), aldolase (158 kDa), catalase (232 kDa), ferritin (440 kDa), and thyroglobulin (669 kDa). The calibration curve of Kav versus log molecular weight was prepared using the equation Kav = (Ve−Vo)/(Vt−Vo,), where Ve = elution volume for the protein, Vo = column void volume, and Vt = total bed volume.
(TIF)

Figure S3 **Sequence alignment of structural homologs of** ***K. pneumoniae*** **NDM-1.** The top 250 structural homologs (Z-score>10), as identified via the DALI server, were clustered (90% sequence identity cut-off). The closest structural homologs are MBLs, followed by class A, C, and D β-lactamases.
(TIF)

Figure S4 **MBL loops.** The representative structures from the 40 structure clusters (shown in Fig. S3), including NDM-1 (molA of 3RKJ) were aligned based on the secondary structure. This alignment reveals variation in the size of five loops contributing amino acid side chains to the active site (labeled Active Site Loops, ASL) in the NDM-1 structure compared to members of structural clusters. NDM-1 shows the largest ASL1 and ASL4 loops in the family.
(TIF)

Figure S5 ***K. pneumoniae*** **NDM-1 is shown with antibiotics that serve as substrates for this MBL modeled into the active site based on coordination of the β-lactam ring to the proposed catalytic site.** To highlight the wide range of conformational states that the compounds occupy in the binding pocket: Biapenem (B), Cefoxitin (C), Faropenem (D), Imipenem (E), Nitrocefin (F) and Tebipenem (G) are shown grouped in A (see kinetic data for these ligands in Fig. S6A–F). The β-lactam ring is shown in black and wide stick representation. For reference, the mobile ASL1 loop is shown in dark gray. Every molecule can be posed within the active site without steric clashing; however, to accomplish this, some unfavorable conformations are adopted.
(TIF)

Figure S6 **Kinetic data for NDM-1 lactamase with selected substrates. A**. Kinetic data for NDM-1 Δ36NY with Nitrocefin. **B**. Kinetic data for NDM-1 Δ36NY with Tebipenem. **C**. Kinetic data for NDM-1 Δ36NY with Imipenem. **D**. Kinetic data for NDM-1 Δ36NY with Biapenem. **E**. Kinetic data for NDM-1 Δ36NY with Cefoxitin. **F**. Kinetic data for NDM-1 Δ36NY with Faropenem.
(TIF)
