# Supplementary figures and images for: Structure of Apo- and Monometalated Forms of NDM-1—A Highly Potent Carbapenem-Hydrolyzing Metallo-β-Lactamase
Source: PLoS One. 2011 Sep 8;6(9):e24621. doi: 10.1371/journal.pone.0024621 (PMC3169612; doi:10.1371/journal.pone.0024621)

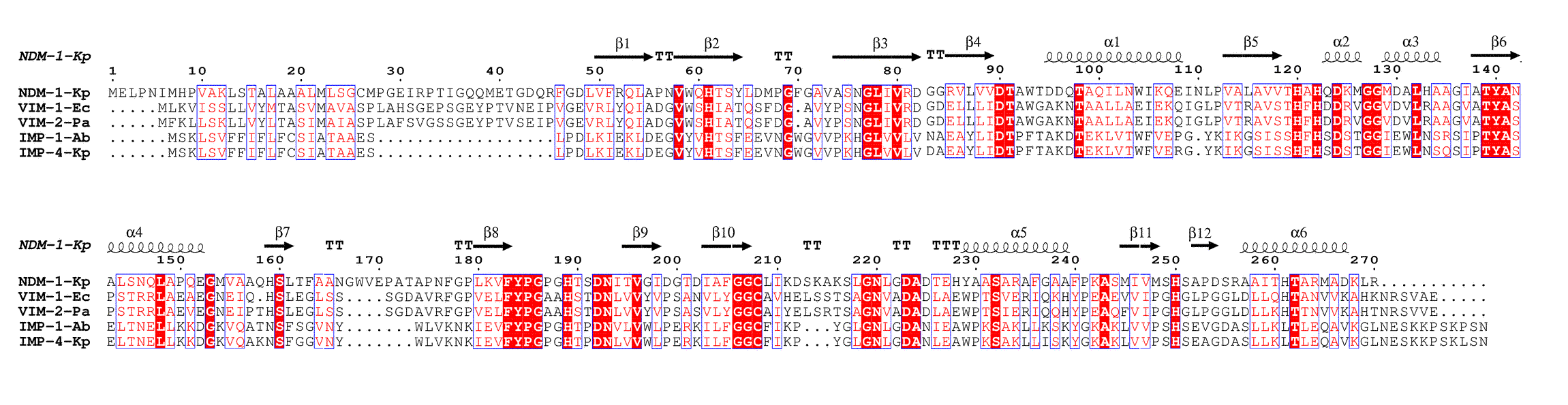

Supplement: Figure S1 — Sequence alignment of selected MBLs. Amino acid sequence of K. pneumoniae NDM-1 was aligned with several MBLs (IMP-1 from Acinetobacter baumannii (gi110350569), IMP-4 from K. pneumoniae (gi|110350569), MBL from B. fragilis (gi|22091056), MBL-2 from B. cereus ATCC 10876 (gi|229191531), VIM-1 protein from Enterobacter cloacae (gi87158436), MBL VIM-11 from P. aeruginosa (gi|49035769), VIM-2 from P. aeruginosa (gi|126571829)) using ClustalX. The ‘T’ denotes turns. The blue boxes denote residues with >0.7 similarity. The Figure was prepared using ESPript 2.2 (http://espript.ibcp.fr/ESPript/ESPript). (TIF) [file pone.0024621.s001.tif]

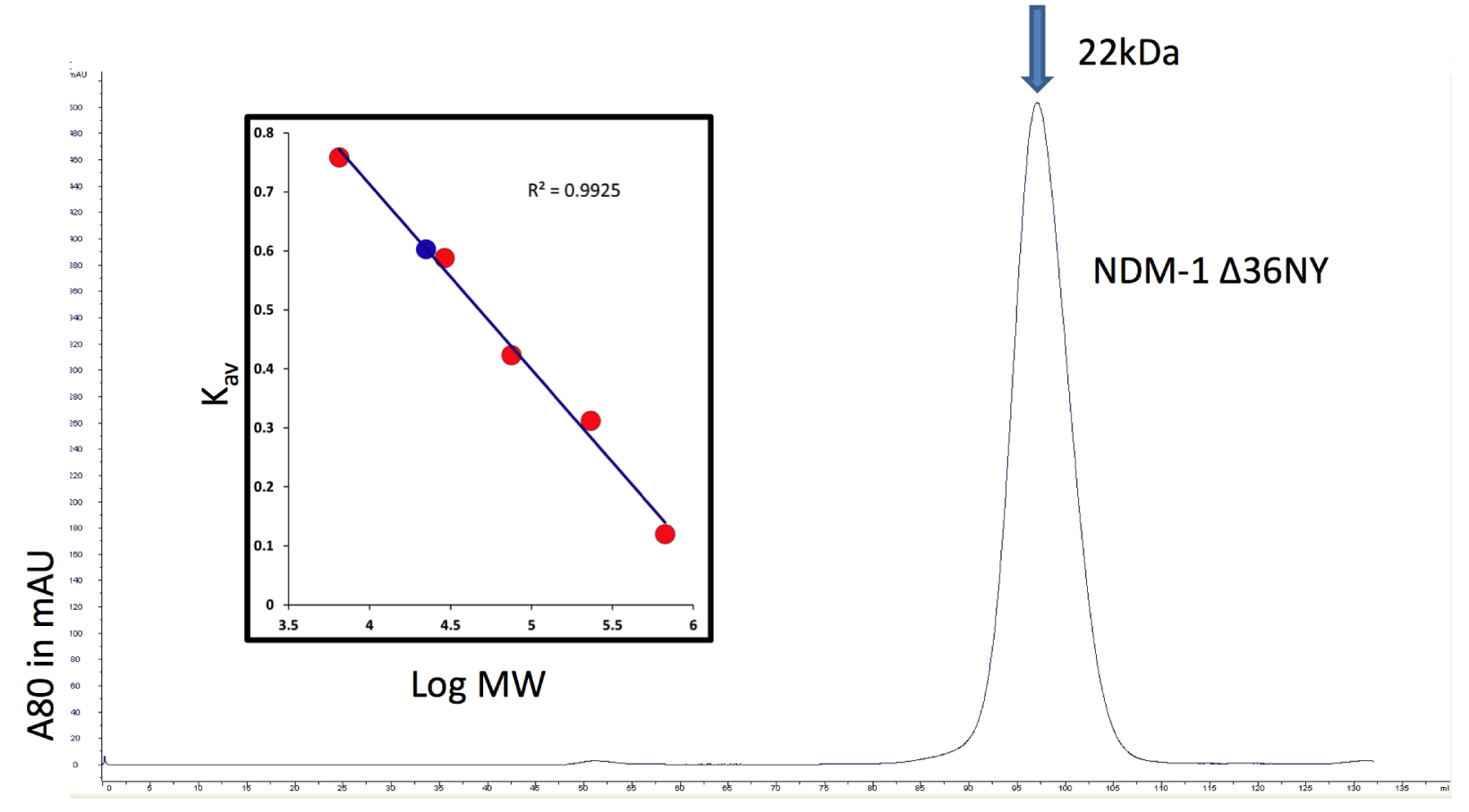

Supplement: Figure S2 — Size exclusion chromatography of native NDM-1 protein using a Superdex 200 GE Healthcare 16/60 column (blue circle). The separation was carried out at 22°C at a flow rate of 2.0 mL/min. The column was calibrated with molecular weight standards (red circles) aprotinin (6.5 kDa), ribonuclease (13.5 kDa), carbonic anhydrase (29 kDa), ovalbumin (43 kDa), conalbumin (75 kDa), aldolase (158 kDa), catalase (232 kDa), ferritin (440 kDa), and thyroglobulin (669 kDa). The calibration curve of Kav versus log molecular weight was prepared using the equation Kav = (Ve−Vo)/(Vt−Vo,), where Ve = elution volume for the protein, Vo = column void volume, and Vt = total bed volume. (TIF) [file pone.0024621.s002.tif]

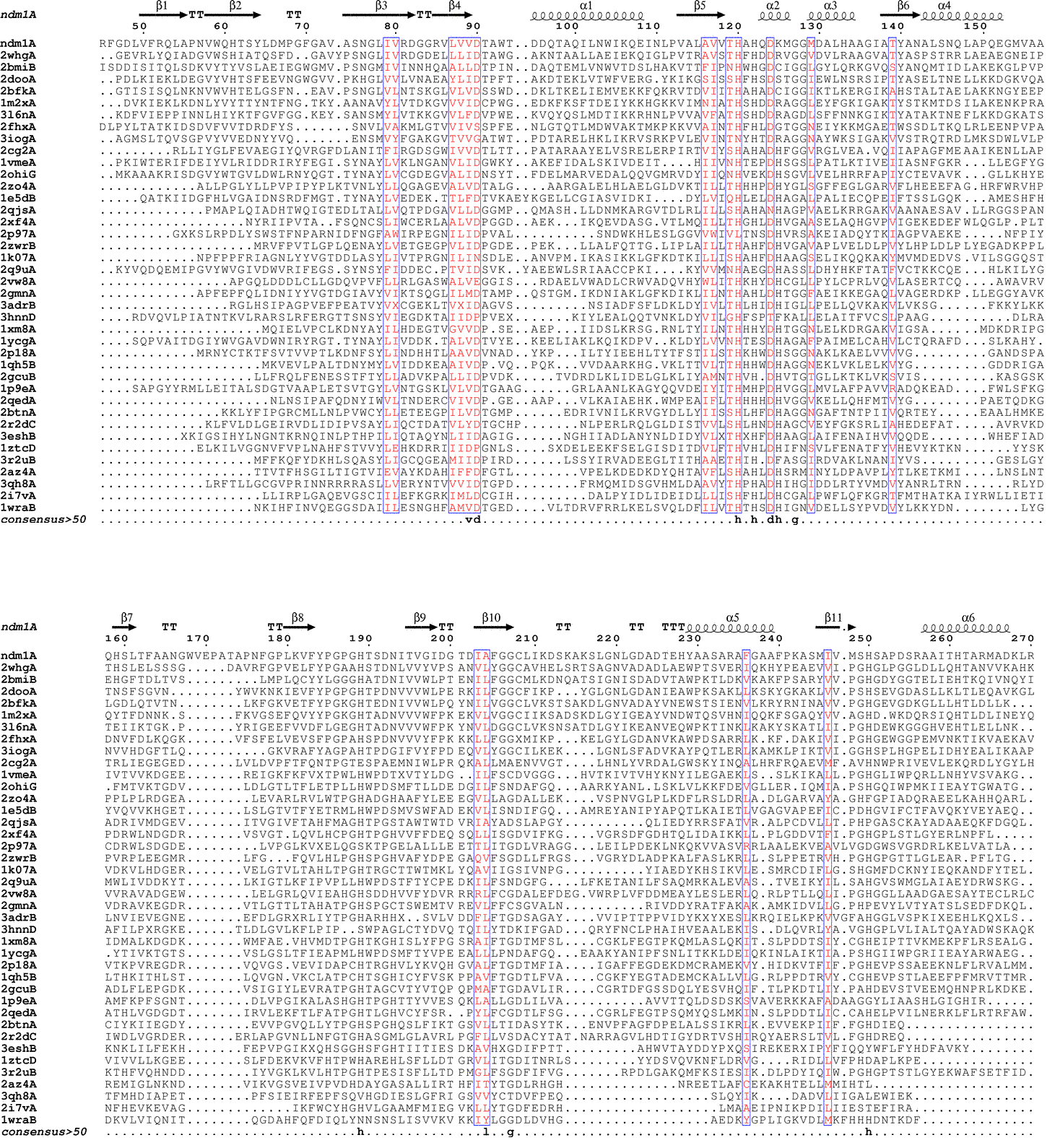

Supplement: Figure S3 — Sequence alignment of structural homologs of K. pneumoniae NDM-1. The top 250 structural homologs (Z-score>10), as identified via the DALI server, were clustered (90% sequence identity cut-off). The closest structural homologs are MBLs, followed by class A, C, and D β-lactamases. (TIF) [file pone.0024621.s003.tif]

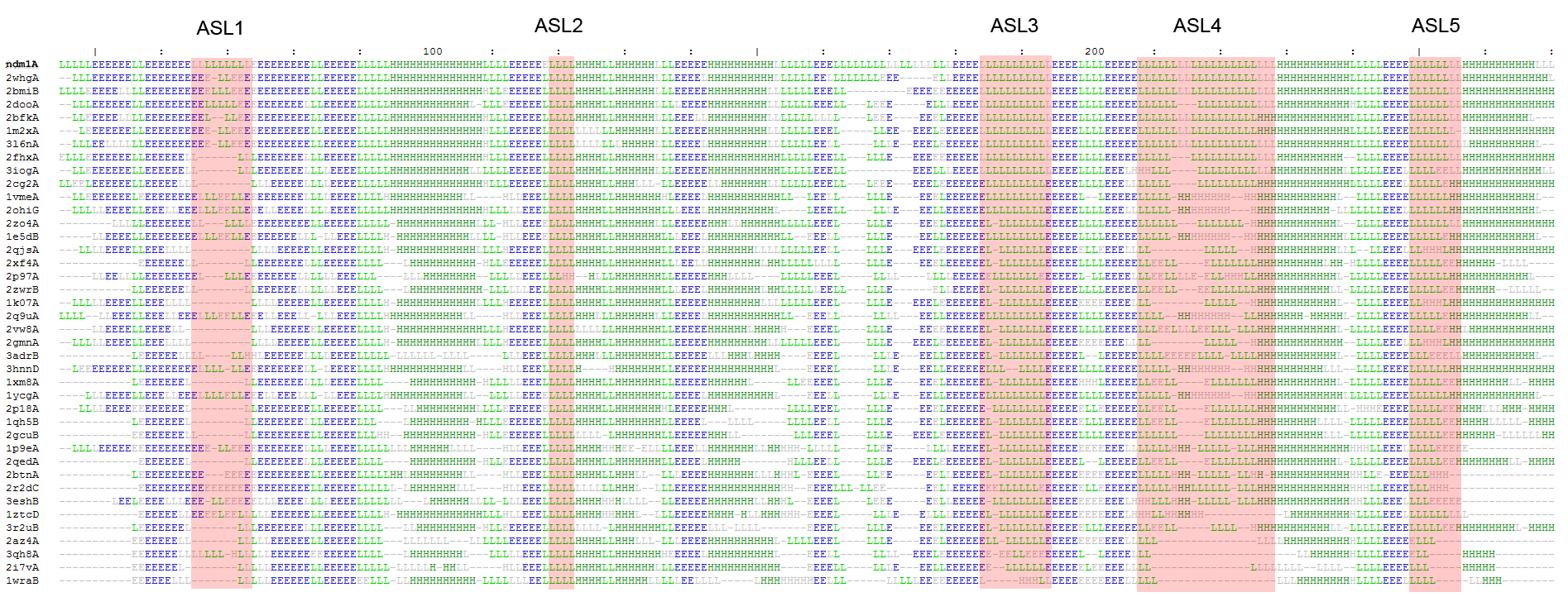

Supplement: Figure S4 — MBL loops. The representative structures from the 40 structure clusters (shown in Fig. S3), including NDM-1 (molA of 3RKJ) were aligned based on the secondary structure. This alignment reveals variation in the size of five loops contributing amino acid side chains to the active site (labeled Active Site Loops, ASL) in the NDM-1 structure compared to members of structural clusters. NDM-1 shows the largest ASL1 and ASL4 loops in the family. (TIF) [file pone.0024621.s004.tif]

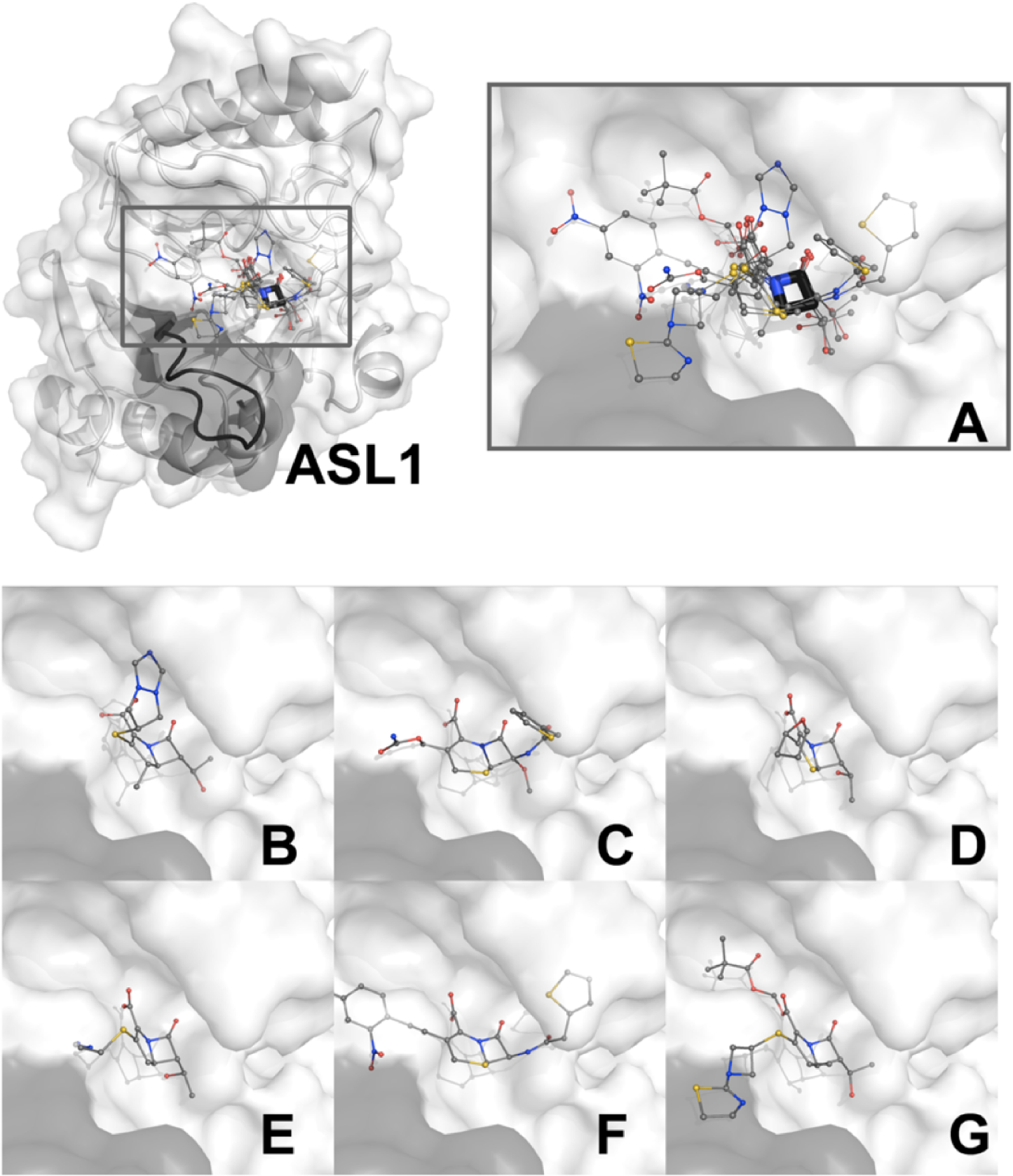

Supplement: Figure S5 — K. pneumoniae NDM-1 is shown with antibiotics that serve as substrates for this MBL modeled into the active site based on coordination of the β-lactam ring to the proposed catalytic site. To highlight the wide range of conformational states that the compounds occupy in the binding pocket: Biapenem (B), Cefoxitin (C), Faropenem (D), Imipenem (E), Nitrocefin (F) and Tebipenem (G) are shown grouped in A (see kinetic data for these ligands in Fig. S6A–F). The β-lactam ring is shown in black and wide stick representation. For reference, the mobile ASL1 loop is shown in dark gray. Every molecule can be posed within the active site without steric clashing; however, to accomplish this, some unfavorable conformations are adopted. (TIF) [file pone.0024621.s005.tif]

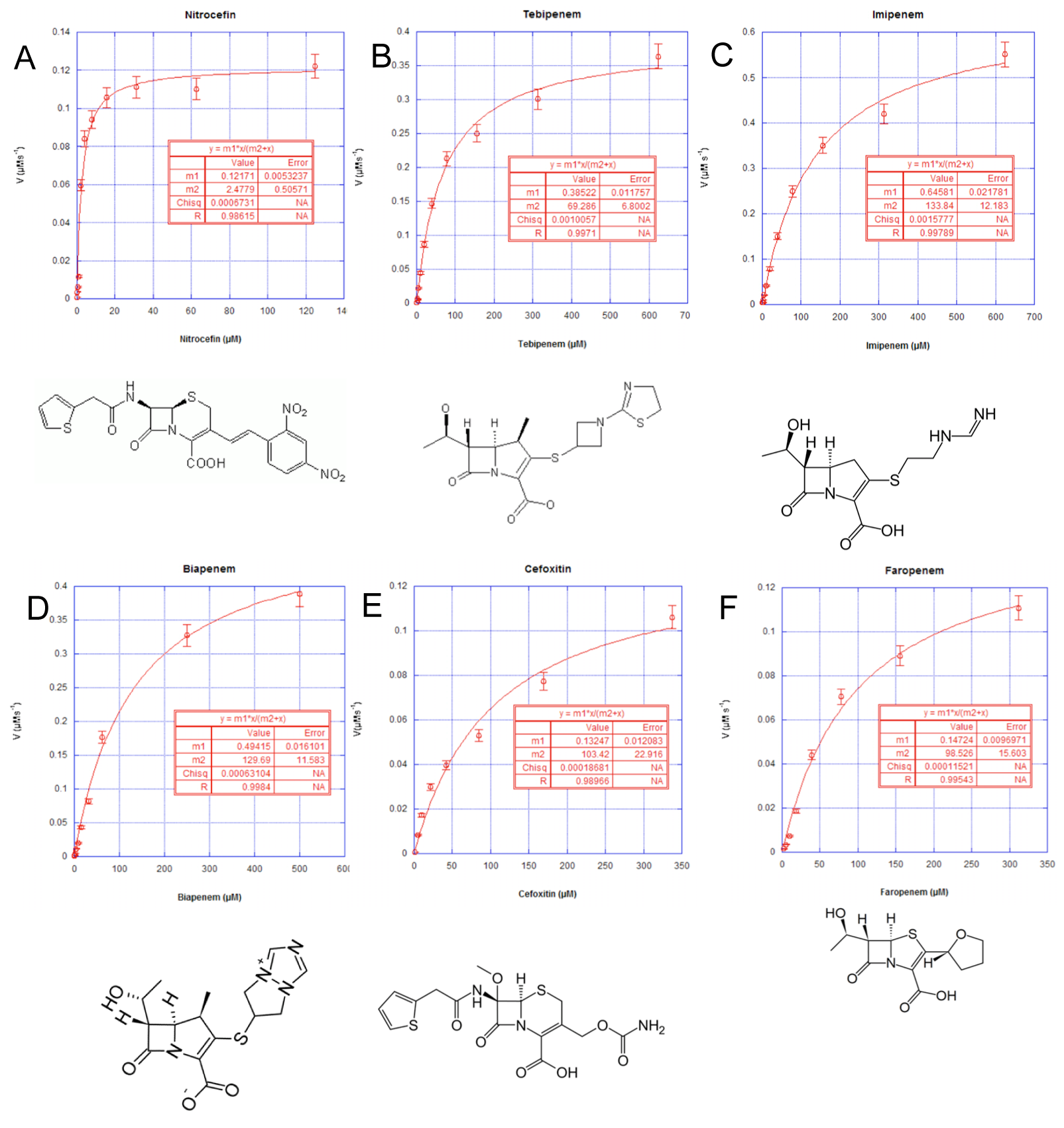

Supplement: Figure S6 — Kinetic data for NDM-1 lactamase with selected substrates. A. Kinetic data for NDM-1 Δ36NY with Nitrocefin. B. Kinetic data for NDM-1 Δ36NY with Tebipenem. C. Kinetic data for NDM-1 Δ36NY with Imipenem. D. Kinetic data for NDM-1 Δ36NY with Biapenem. E. Kinetic data for NDM-1 Δ36NY with Cefoxitin. F. Kinetic data for NDM-1 Δ36NY with Faropenem. (TIF) [file pone.0024621.s006.tif]
